# Intraoperative transradial angiography augments safe hysterectomy for uterine fibroids in the setting of ambiguous arterial anatomy: a case report

**DOI:** 10.1186/s13256-019-2154-0

**Published:** 2019-07-29

**Authors:** Akhil A. Chandra, Anthony N. Grieff, Adrian C. Balica, William E. Beckerman

**Affiliations:** 1 Division of Vascular Surgery and Endovascular Therapy, Rutgers Robert Wood Johnson School of Medicine, One Robert Wood Johnson Place, MEB 541, New Brunswick, NJ 08901 USA; 2 Department of Obstetrics, Gynecology and Reproductive Sciences, Rutgers Robert Wood Johnson School of Medicine, New Brunswick, NJ USA

**Keywords:** Uterine fibroids, Uterine artery embolization, Uterine fibroid embolization, Transradial access, UAE, UFE, TRA

## Abstract

**Background:**

Transfemoral access is the traditional gold standard for uterine artery angiography; however, transradial access is gaining in popularity because of its decreased complication profile and patient preference. We present a case of a patient who underwent successful total abdominal hysterectomy for symptomatic uterine fibroids with ambiguous pelvic vasculature that would have been otherwise aborted if it were not for intraoperative transradial access angiography.

**Case presentation:**

A 52-year-old Caucasian woman presented to her gynecologist for an elective total abdominal hysterectomy and bilateral salpingo-oophorectomy. During preoperative imaging, a 15-cm mass consistent with a uterine fibroid was identified, and the patient’s gynecologist decided to treat her with surgical resection, given the fibroid’s size. The procedure was halted upon discovery of a complicated vascular plexus at the fundus of the uterus, and an intraoperative vascular consult was requested. The vascular operator used a transradial access to perform pelvic angiography in real time to identify the complicated pelvic vasculature, which allowed the gynecologist to surgically resect the uterine fibroid. The patient was discharged on postoperative day 4 without any complications.

**Conclusions:**

Intraoperative imaging is a useful technique for the identification of complicated anatomical structures during surgical procedures. The successful outcome of this case demonstrates an additional unique benefit of transradial access and highlights an opportunity for interdisciplinary collaboration for management of complicated surgical interventions.

## Background

Uterine artery angiography and embolization has emerged as an effective alternative to open and laparoscopic surgery for benign uterine fibroids with the benefits of lower cost, shorter hospital stay, and faster recovery time [1]. Transfemoral arterial access (TFA) is the most common approach; however, in recent years, transradial arterial access (TRA) has emerged as an alternative. Much of the reason for this stems from a well-documented track record of success in coronary interventions since the 1990s [2]. The main advantages of TRA are fewer access site complications, reduced cost, and increased patient comfort [2]. An often overlooked aspect of TRA is that the arm remains readily accessible and free of the standard abdominal operative field, whereas TFA approaches are technically challenging and pose increased risk of contamination during open abdominal and pelvic procedures.

We report a case of a patient who presented to our clinic for an elective total abdominal hysterectomy and bilateral salpingo-oophorectomy (TAH-BSO). The patient’s procedure was halted upon the discovery of ambiguous pelvic vasculature at the fundus of the uterus. The gynecologic operator on the case requested an intraoperative consultation from the vascular service at our institution. A decision was made by our vascular service to perform pelvic angiography via a TRA, an access point that allowed both operators to concurrently work on the case intraoperatively to resolve the complication. Consequently, this case proved to be a novel collaboration between the gynecologic and vascular services at our institution and represented a useful opportunity to enhance patient outcomes through the use of TRA.

## Case presentation

A 52-year-old Caucasian woman presented with discomfort and menorrhagia to her gynecologist for an elective TAH-BSO. Her past medical history was significant for hypertension, hypothyroidism, nephrolithiasis, uterine fibroids, and cervical squamous intraepithelial neoplasia. She reported a 28-day menstrual cycle with 8 days of heavy menstrual bleeding accompanied by mild cramping. Her past surgical history was significant for a wisdom tooth extraction. She had a 0.25–pack-year smoking history and denied any prior alcohol or recreational drug use. The patient lives with her mother and is currently employed as a hair stylist. She denied knowledge of any sexually transmitted infections and further stated that she had not been sexually active for the past 20 years. Her prior medication history included an adult aspirin 81 mg daily, carvedilol 25 mg twice daily, hydrochlorothiazide 12.5 mg daily, and omeprazole 40 mg daily. While admitted in our hospital, she was administered Percocet (oxycodone-acetaminophen) 5 mg every 4–6 hours, Tylenol (acetaminophen) 1000 mg every 4–6 hours, and Zofran (ondansetron) 4 mg every 4–6 hours. Her vital signs upon admission were blood pressure 136/81 mmHg, heart rate 69 beats/minute, respiration rate 12 respirations/minute, and 100% O_2_ saturation, and she was afebrile. Results of her laboratory tests are shown in Table 1.

**Table 1:** Laboratory test results

Test (normal range)	Results
Glucose (70–100 mg/dl)	87
BUN (6–23 mg/dl)	9
Creatinine (0.5–1.2 mg/dl)	0.6
Calcium (8.6–10.4 mg/dl)	8.9
Sodium (136–145 mmol/L)	140
Potassium (3.5–5.0 mmol/L)	3.8
Chloride (98–108 mmol/L)	102
Total CO_2_ (22.0–30.0 mmol/L)	26.1
Anion gap (7.0–17.0 mEq/L)	12.0
Magnesium (1.8–2.5 mg/dl)	1.9
WBC (4.0–10.0 × 10^3^/μl)	6.3
Hemoglobin (11.9–15.1 g/dl)	8.0
Hematocrit (36.7–44.7%)	23.7
Platelet count (140–440 × 10^3^/μl)	330

*BUN* Blood urea nitrogen, *WBC* White blood cell count

During her physical examination, the patient appeared well-nourished. Upon general inspection, she was normocephalic and atraumatic. Her pupils were reactive to light bilaterally with functioning extraocular motor movements and clear conjunctiva and sclera. Her tympanic membranes were intact, and she displayed grossly normal hearing. No thyromegaly or abnormal cervical lymphadenopathy on her neck was discovered. Her lungs were clear bilaterally upon auscultation. Her chest was nontender, and auscultation revealed S1/S2 with regular rate and rhythm and nondisplaced point of maximal impulse. Inspection of the abdomen revealed no signs of hepatosplenomegaly, umbilical hernias, or abnormal masses. Her bowel sounds were auscultated in all quadrants and were normal. Her pulses were strong and even bilaterally in all extremities. She was alert and oriented to person, time, and place. She was cooperative with normal mood, attention span, and concentration. Neurological examination revealed that her cranial nerves I–XII were grossly intact.

On preoperative magnetic resonance imaging, she was found to have a 15-cm mass consistent with a uterine fibroid that appeared to be posteriorly fixed to the retroperitoneum with intimate involvement of the pelvic vasculature that was only amenable to open surgical resection because of its size.

In the operating theater, a midline abdominal incision was made, followed by ureterolysis. The procedure was halted after encountering a large vascular plexus at the uterine fundus, concerning for an aberrant vascular pedicle. Historically, the case would have been aborted so that the patient could undergo either catheter-based or computed tomographic angiography followed by potential return to the operating room. However, a recent collaborative effort at our institution between vascular surgery and gynecology in the management of uterine fibroids led to an intraoperative vascular consult request. A decision was made to perform intraoperative transradial pelvic angiography with the intent, if necessary, to embolize or temporarily balloon-occlude any feeding vessels or main uterine arteries to allow for safe surgical resection.

**Fig. 1:**
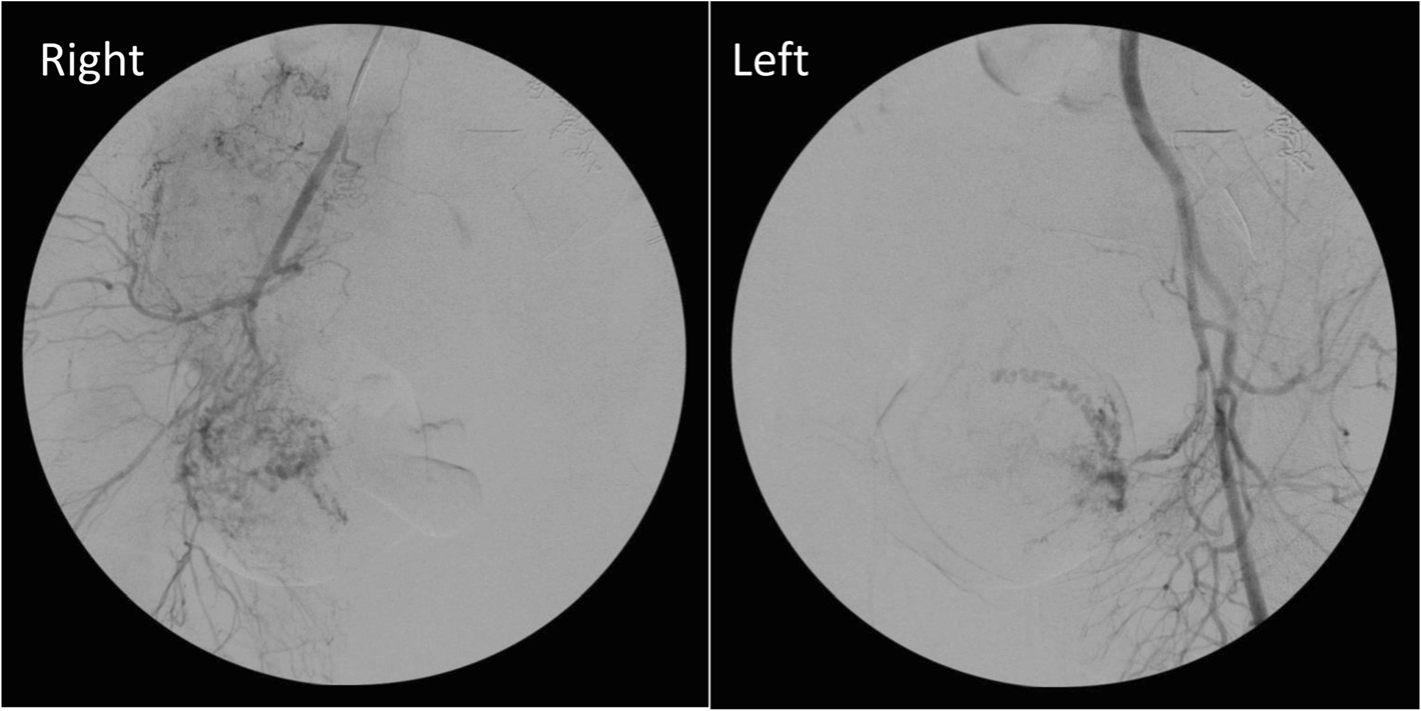
Bilateral internal iliac artery angiography

**Fig. 2:**
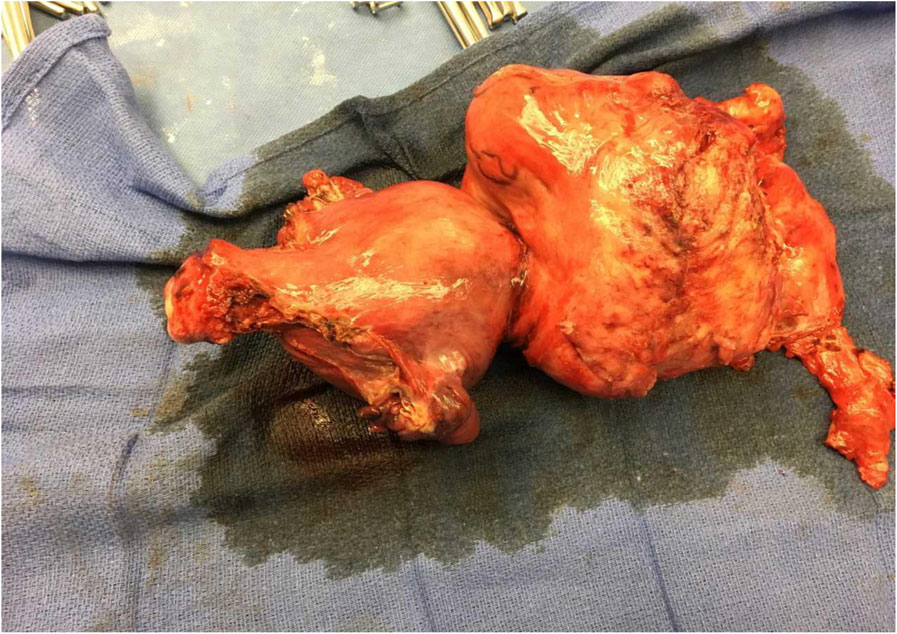
Gross anatomy of excised uterine fibroid

The operative field was covered with a sterile drape, and a fluoroscopy C-arm was positioned. The patient’s left arm was abducted on an arm board, and a Barbeau test [3] was performed to confirm adequate ulnar perfusion of the hand. Under ultrasound guidance, the left radial artery was accessed. Given the unplanned nature of this procedure and the patient’s open abdomen, the typical radial cocktail of heparin and vasodilators was not administered, and a 4/5-French Glidesheath Slender sheath (Terumo Medical Co., Somerset, NJ, USA) was inserted with a saline side flush on a pressure bag to minimize complications of vasospasm or thrombosis. A diagnostic catheter was advanced into the abdominal aorta over a Bentson wire, and a diagnostic angiogram of the bilateral iliac tree demonstrated normal vascular anatomy. The bilateral internal iliac arteries and subsequently uterine arteries were then selected to determine the absence of large aberrant vessels (Fig. 1). Following this, the gynecology team reentered, and the surgical resection was completed without complications (Fig. 2). To ensure the availability of quick intervention in the event of vascular injury, the radial sheath was maintained for the remainder of the case.

Just prior to the patient being taken to the postoperative anesthesia unit, the sheath was removed and a transradial TR Band (Terumo Medical Co.) was inflated at the puncture site to achieve patent hemostasis. Two hours later, after successful hemostatic placement was achieved, the TR Band was removed, and the patient had no signs of hand ischemia or radial artery thrombosis. She was discharged to home on postoperative day 4 without complications. At her 6-month follow-up visit, the patient was doing well without any significant complaints.

## Discussion

We report a case of a 52-year-old woman who presented to her gynecologist for an elective TAH-BSO. Her preoperative imaging revealed a 15-cm uterine fibroid that required surgical resection. The procedure was halted upon discovery of a complicated vascular plexus at the fundus of the uterus, and an intraoperative vascular consult was requested. The vascular service at our institution used a TRA to perform intraoperative pelvic angiography and identified the complicated pelvic vasculature so that the gynecologist could operate on it. Prior literature on TRA has focused on its rates of efficacy, number of adverse complications, and safety profile in relation to TFA (Table 2). However, there has not been work completed exploring its usefulness as an interdisciplinary tool. The present case report examines the use of TRA within the setting of an intraoperative consultation and highlights a novel team-based approach.

**Table 2:** Comparison of transradial and transfemoral arterial access

	Transradial access (TRA)	Transfemoral access (TFA)
Entry point	Radial artery	Femoral artery
Founded	1989	~1960s
Advantages	• Superficial artery for easier visualization • Readily compressible • Less susceptible to effects of thrombosis due to dual blood supply of the hand • Hemostasis achieved without the introduction of a vascular closure device (transradial band used instead) • Shorter procedural time • Faster recovery time with immediate ambulation • Lower procedural cost • Studies indicate 100% technical success rate at 1-month follow-up • Fewer bleeding complications • Lower rates of morbidity and mortality	• Many trained physicians comfortable with this approach • Large arterial diameter • Long history of success
Disadvantages	• Learning curve that can lead to vascular complications • Small radial artery diameter • Radial artery spasm, radial artery occlusion, and/or forearm hematoma	• Higher risk of hemorrhage • Longer time necessary until discharge • Greater procedural cost • Femoral artery provides the only blood supply to the leg; occlusion can have severe consequences

The uterine artery is the primary blood supply to the uterus and supports physiological processes including alteration of endometrium during menstruation and nourishing the growing uterus during pregnancy [4]. Not all symptomatic patients requiring intervention are good candidates for minimally invasive myomectomy or primary uterine fibroid embolization. They often require open surgical management, most commonly hysterectomy [4, 5]. In the setting of enlarging uterine fibroids, the principal vascular supply by the uterine artery becomes distorted and tortuous, leading to difficult identification, and known common complications of hysterectomy include blood loss and vascular injury [6]. As seen in our patient’s case, angiography is an invaluable tool for safely identifying ambiguous anatomy and confirming the presence or absence of aberrant vessels in order to achieve safe surgical resection.

Because our patient was midway through an exploratory laparotomy, TRA was superior to TFA, given that the groin was not easily accessible owing to surgical draping. This method also provided the vascular and gynecological operators their own respective surgical fields. Furthermore, it should be noted that we do not believe there were any specific advantages lost by using TRA as compared with TFA. In addition, separation of operative fields reduced the risk of cross-contamination and allowed the vascular operators and nursing staff a technically easier site to cannulate and manage. TRA also avoided the need to modify the operative drape to provide the surgical operators access to the groin. This alleviated the time required to redrape the abdomen after angiography, thus reducing operative time. Furthermore, TRA is efficient in terms of time, motion, and supplies because the vessels are caudally oriented and easily cannulated from a completely antegrade approach. This is in direct contrast to the retrograde access of the internal iliac artery required with TFA, which often requires significant time in the setting of tortuous vessels.

Some complications associated with TRA include radial artery spasm, radial artery occlusion, access site hematoma, forearm hematoma, and failed access owing to poorly guided support [7, 8]. In particular, radial artery spasm has been shown to be accountable for up to 38% of all TRA failures [8]. The steep learning curve for TRA is often the culprit of these adverse complications and can be mitigated through proper technique and experience [8]. The use of shorter hydrophilic coated sheaths, heparin, and spasmolytic vasodilatory agents (verapamil, diltiazem) has been shown to reduce the incidence of radial artery spasm [7]. Likewise, using a smaller catheter can reduce the incidence of radial artery occlusion, especially in patients with a smaller radial artery diameter [9]. Most important, the risk of postprocedural radial artery thrombosis can be greatly minimized by ensuring nonocclusive “patent” hemostasis [7, 10].

At the end of the procedure, a TR Band was placed on our patient’s left wrist after palpation of the distal radial artery pulse. The TR Band was removed safely and mitigated the requirement to hold direct pressure or use an invasive closure device as would be required in a transfemoral case, thus decreasing case duration.

When performed by an experienced surgeon, the benefits of TRA outweigh the potential downside, and, in our patient’s case, TRA was the most practical intraoperative modality. In patients undergoing invasive treatments, TRA has been found to have significantly lower rates of major hemorrhage and mortality than the TFA [11, 12], which is likely related to TRA’s superior complication profile. A retrospective study of TRA in uterine artery embolization by an experienced interventional radiology group found a 100% technical success rate with no major adverse complications recorded, including no radial artery occlusions at 1-month follow-up. Furthermore, a retrospective study completed by an interventional radiology group and gynecologic group also found that TRA had a 100% technical success rate in uterine fibroid embolization and determined that TRA (60.3 minutes) required less procedural time than TFA (72.4 minutes) [13]. Finally, it should be noted that intraoperative vascular consultation in TAH-BSO is a rare event and likely is unpreventable because it is dependent on the surgeon’s clinical assessment and intraoperative findings.

## Conclusions

We present a case of a patient who underwent successful total abdominal hysterectomy for symptomatic uterine fibroids, which would have been otherwise aborted secondary to ambiguous pelvic vasculature if it were not for intraoperative TRA. The successful outcome of this case demonstrates an additional unique benefit of TRA and highlights potential opportunities for future interdisciplinary collaboration.

## Data Availability

Not applicable.

## Abbreviations

TAH-BSO: Total abdominal hysterectomy and bilateral salpingo-oophorectomy
TFA: Transfemoral access
TR: Transradial
TRA: Transradial access
